# Identifying Cerebral Spinal Fluid Leaks by Catheter Targeted Dynamic Myelograms with Directed Intrathecal Enhancement Tomography (DIET) Technique

**DOI:** 10.1161/SVIN.124.001683

**Published:** 2025-03-23

**Authors:** Thomas Snyder, Matthew Tangel, Haralabos Zacharatos, Brian T. Jankowitz

**Affiliations:** ^1^ Department of Neurology, Hackensack Meridian Health JFK University Medical Center Edison NJ; ^2^ Department of Radiology, Hackensack Meridian Health JFK University Medical Center Edison NJ; ^3^ Department of Neurosurgery, Hackensack Meridian Health JFK University Medical Center Edison NJ

A man in his 30s presented with classical signs and symptoms of a cerebral spinal fluid (CSF) leak. He underwent magnetic resonance imaging of the total spine that showed epidural contrast from C3 to C9 levels in the anterior epidural space followed by a digital subtraction myelography in prone position which did not locate the leak well enough to consider surgical therapy. Next, he underwent a catheter targeted dynamic myelogram by directed intrathecal enhancement tomography (DIET) technique, which detected a focal ventral epidural leak at the level of C6 left of the midline.

A woman in her 50s who had a retrosigmoid craniotomy presented with rhinorrhea and moisture in the ear cavity. She was referred to help discern if the CSF leak was from the petrous or mastoid part of the temporal bone. She underwent a catheter targeted dynamic myelogram using DIET technique, which detected the focal CSF leak in the left mastoid air cell.

Both procedures were performed by introducing an INRAD needle (20 “GA x 5 ⅞”) (INRAD Inc., Kentwood, MI, USA) between the L2/L3 space with the patient in prone position. The INRAD needle was selected for its extra length, allowing for a shallower trajectory, facilitating a natural entry into the thecal sac.

A nitrex wire (Medtronic, Minneapolis, MN, USA) was advanced into the intrathecal space through the INRAD needle. The needle was then removed. The nitrex wire was used as it is less likely to shear compared to other wires when the needle tip comes into contact with the wire.

The inner dilator of a Merit MAKNV introducer system (Merit Medical Systems, Salt Lake City, UT, USA) was advanced over the wire. This creates a tract large enough for a microcatheter with an inner diameter of 0.021“ and yet sturdy enough to easily traverse the lumbar fascia and ligamentous complex. The nitrex wire was then exchanged for a V18 wire (Boston Scientific, Marlborough, MA, USA) to provide enough support for advancement of a catheter through the lumbar fascia bareback. The inner dilator of the Merit MAKNV was exchanged over the wire for a TruSelect microcatheter. Once the TruSelect microcatheter (Boston Scientific, Marlborough, MA, USA) was confirmed to be in the intrathecal space, the V18 wire was removed. and a 0.014” synchro wire (Stryker, Kalamazoo, MI, USA) was advanced with the catheter. The catheter was never advanced through the inner lumen of a larger access needle or trocar, thus reducing tissue damage and dural hole size. In our cases, the catheter was advanced to the superior endplate of C7 and to the level of the foramen magnum. A 3‐way stopcock was attached to the end of the microcatheter. Redundant tubing was secured to the patient's lower back with a large Tegaderm (Solventum, Maplewood, MN, USA). A 20 cc syringe of sterile saline was attached to 1 hub to both engorge the target location with fluid to augment the CSF leak and to chase contrast that is stagnating too proximal to the target location if Trendelenburg maneuvers do not work adequately. A 10 cc syringe of contrast was attached to the other hub (Figure 1). The system was now closed and sterile allowing for safe transportation.

**Figure 1:**
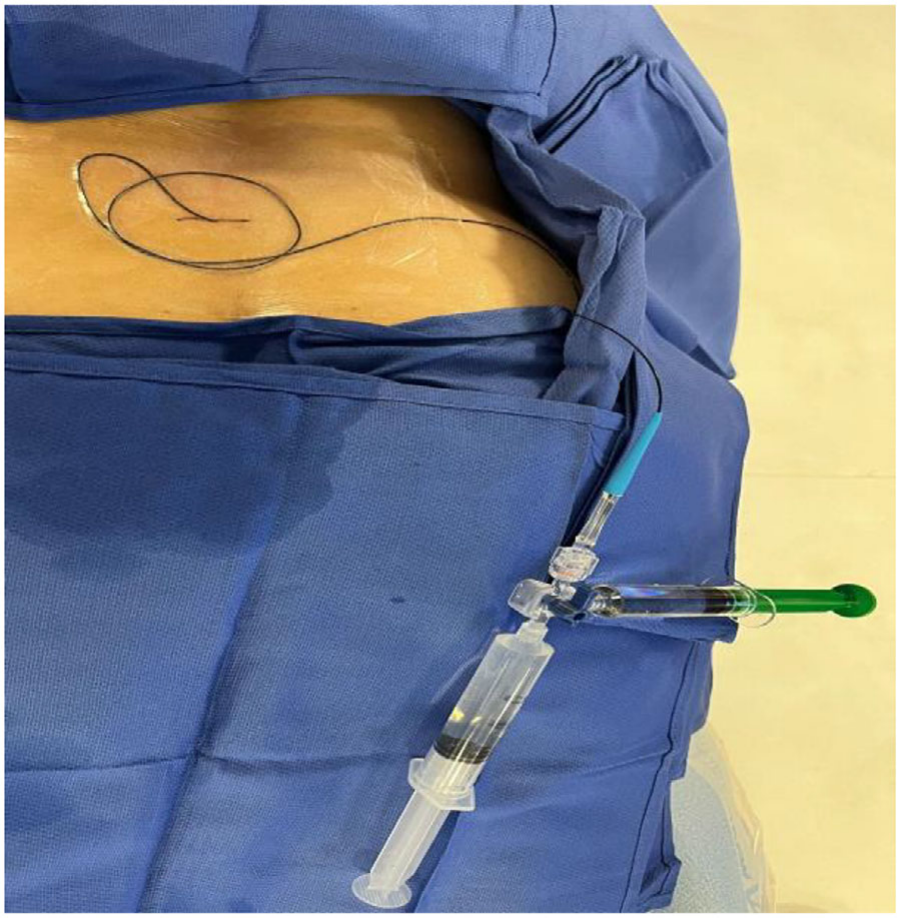
Image of patient being transported from operating table to scanner for dynamic computed tomography myelography component of procedure.

Patients were transferred to the computed tomography scanner in a prone position with a pillow slightly extending the neck. This slows the advancement of contrast into the supratentorial space to avoid the rare but documented reports of seizures. Then, 10 cc of injectable 0.9% saline was administered by the operator through a 20 cc syringe. Sequences were performed before and after the administration of 10 mL Omnipaque‐300 (GE HealthCare, Chicago, IL, USA) through the 10 cc syringe.

In both cases, the focal point of the CSF leak was able to be identified without causing an additional CSF leak or seizure.

Despite the use of advanced imaging, locating the origin of a CSF leak still has many limitations. Currently, the sensitivity of digital subtraction myelography to detect a CSF leak varies from 15%–80% depending on technique.^1^, ^2^, ^3^, ^4^

We describe the DIET technique where a microcatheter was directed to a targeted level and a dynamic computed tomography myelography was performed (Figures 2 and 3). Access into the intrathecal space between L2/L3 was obtained and a microcatheter was advanced for targeted contrast bolus administration to identify the origin of CSF leak (Figure 4). This technique is reproducible, simplifies access, avoids high cervical punctures, reduces radiation, and allows for administration of contrast to an area that is suspected to be the culprit of a CSF leak.

**Figure 2:**
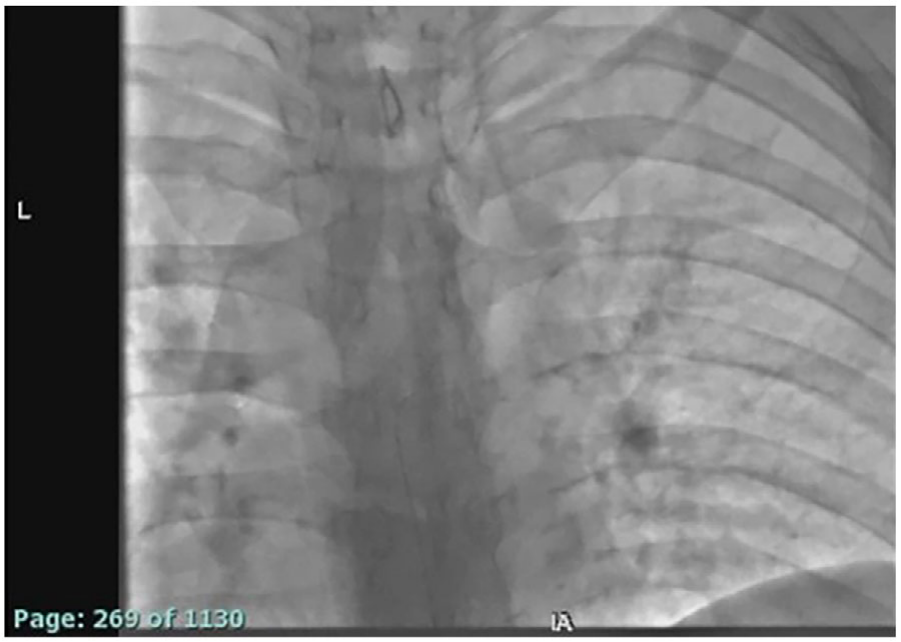
Video demonstrating the microwire and microcatheter selecting nerve roots while being advanced through the thecal sac to cervical vertebra 7.

**Figure 3:**
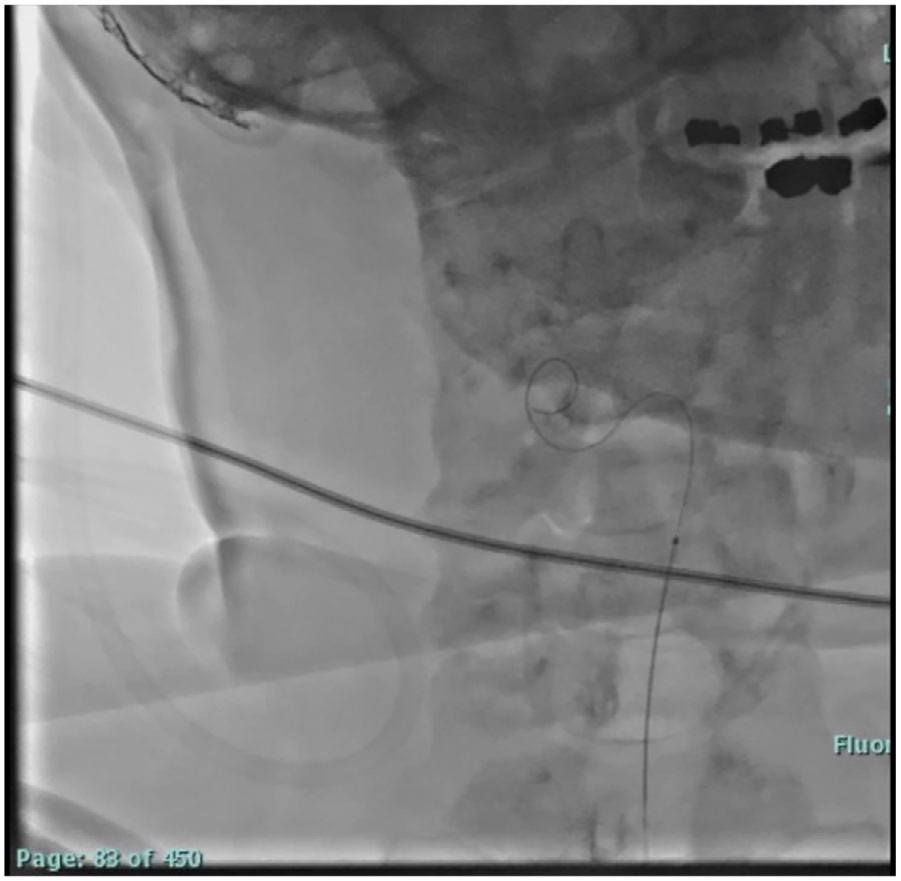
Video demonstrating microwire and microcatheter being advanced through thecal sac to the level of the foramen magnum.

**Figure 4:**
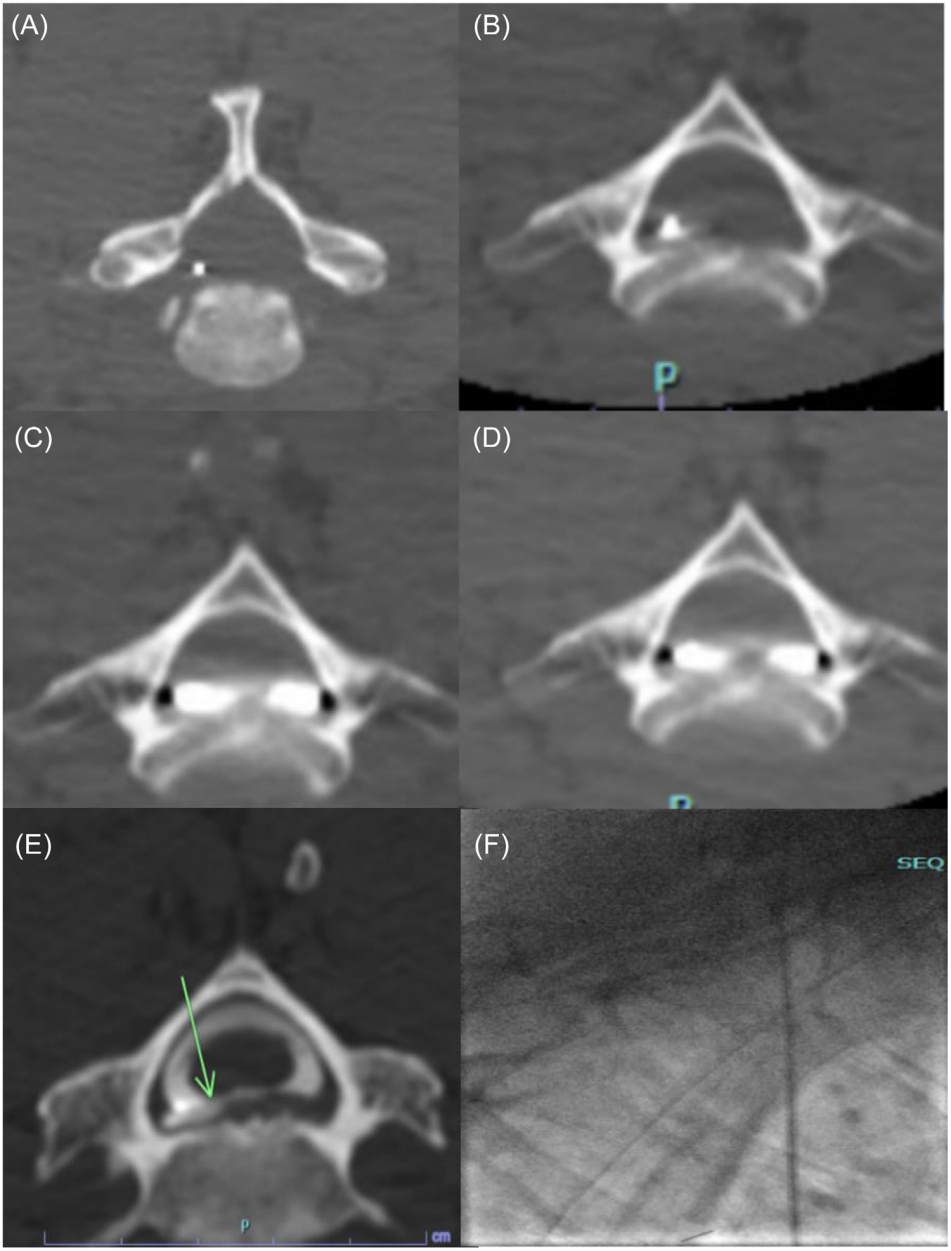
**Dynamic CT myelogram demonstrating the opacification of the thecal sac at the distal tip of the microcatheter representing how quickly opacification evolves over seconds potentially hindering the ability to detect site of CSF leak**. **A,** displays the site before injection; **B**, **C**, and **D** were captured at 30 second intervals after contrast injection. **E,** shows the focal CSF leak origin compared with (**F**), which shows best cervical visualization during DSM in prone position. CSF indicates cerebral spinal fluid; CT, computed tomography; and DSM, digital subtraction myelography.

It was noted that the catheter could be tracked through the intrathecal space using a standard 0.014' wire that favorably recapitulated intravascular access, thereby expanding targeted delivery to every nerve root sleeve and dorsal or ventral dural tears. This could expand application to direct nerve root sleeve injections for diagnosis of CSF leaks with the potential to consider intradural therapeutic options.

## Data Availability

The data used in this study are available upon reasonable request from the corresponding author. Data have been anonymized to respect patient privacy.
